# Analyses of *pp*, *Cu*–*Cu*, *Au*–*Au* and *Pb*–*Pb* Collisions by Tsallis-Pareto Type Function at RHIC and LHC Energies

**DOI:** 10.3390/e24091219

**Published:** 2022-08-30

**Authors:** Li-Li Li, Muhammad Waqas, Muhammad Ajaz, Ahmed M. Khubrani, Hui Yao, Muhammad Adil Khan

**Affiliations:** 1 Department of Basic Sciences, Shanxi Agricultural University, Jinzhong 030801, China; 2 School of Nuclear Science and Technology, University of Chinese Academy of Sciences, Beijing 100049, China; 3 Department of Physics, Abdul Wali Khan University Mardan, Mardan 23200, Pakistan; 4 Department of Physics, Faculty of Science, Jazan University, Jazan 45142, Saudi Arabia; 5 Department of Physics, Islamia College Peshawar, Peshawar 25120, Pakistan

**Keywords:** identified, strange, effective temperature, mass-dependent, transverse momentum spectra, mean transverse momentum, 12.40.Ee, 13.85.Hd, 25.75.Ag, 25.75.Dw, 24.10.Pa

## Abstract

The parameters revealing the collective behavior of hadronic matter extracted from the transverse momentum spectra of $\pi^{+}$, $\pi^{-}$, $K^{+}$, $K^{-}$, *p*, $\bar{p}$, $K_{s}^{0}$, Λ, $\bar{\Lambda }$, Ξ or $\Xi^{-}$, ${\bar{\Xi }}^{+}$ and Ω or ${\bar{\Omega }}^{+}$ or $\Omega +\bar{\Omega }$ produced in the most central and most peripheral gold–gold (Au–Au), copper–copper (Cu–Cu) and lead–lead (Pb–Pb) collisions at 62.4 GeV, 200 GeV and 2760 GeV, respectively, are reported. In addition to studying the nucleus–nucleus (AA) collisions, we analyzed the particles mentioned above produced in pp collisions at the same center of mass energies (62.4 GeV, 200 GeV and 2760 GeV) to compare with the most peripheral AA collisions. We used the Tsallis–Pareto type function to extract the effective temperature from the transverse momentum spectra of the particles. The effective temperature is slightly larger in a central collision than in a peripheral collision and is mass-dependent. The mean transverse momentum and the multiplicity parameter ($N_{0}$) are extracted and have the same result as the effective temperature. All three extracted parameters in pp collisions are closer to the peripheral AA collisions at the same center of mass energy, revealing that the extracted parameters have the same thermodynamic nature. Furthermore, we report that the mean transverse momentum in the Pb–Pb collision is larger than that of the Au–Au and Cu–Cu collisions. At the same time, the latter two are nearly equal, which shows their comparatively strong dependence on energy and weak dependence on the size of the system. The multiplicity parameter, $N_{0}$ in central AA, depends on the interacting system’s size and is larger for the bigger system.

## 1. Introduction

The study of identified and strange particles in high-energy collisions is fundamental. The former allows the disquisition of the particle production mechanisms in a superhot and dense nuclear matter and explores the features of the quark–gluon plasma (QGP). On the other hand, the strange particles are a magnificent probe to identify the phase boundary of the onset of the deconfinement. The transverse momentum ($p_{T}$) spectra of identified particles are one of the pillars of significant discoveries in high-energy physics [1,2,3,4]. According to [5,6], the shape of the spectra is sensitive to the dynamic of nucleus–nucleus collisions and may be used to get the radial flow and the temperature at freeze-out. Additionally, the hadrons with strange content are argued to have smaller hadronic interaction cross-sections and may decouple earlier from the system compared to nonstrange particles [1,5,6]. In this way, the hadrons with strange content would carry direct information from the collisions at an earlier stage without dilution due to hadronic scattering at a late stage. The particles with a lower cross-section interaction are supposed to freeze out early [7,8]. However, other studies [9,10] claim that the decoupling of the particles depends on their mass such that massive particles decouple early from the system. In other works [11,12,13,14,15,16], the decoupling scenarios of different particles are different, including the single decoupling scenario in which all particles are decoupled at the same time, the double-decoupling scenario in which strange and nonstrange particles are decoupled separately, and each particle’s multiple decoupling scenarios for decoupling from the system, respectively. This is an open question up to now in the community. There are two types of freeze-out/decoupling after the fireball expansion. The system cools down as it expands, and the quarks and gluons become reconfined and hadronized. Two other transitions, chemical freeze-out and kinetic freeze-out, happen along the way. The former occurrence is very close to the phase transitions line. It is marked by the system’s temperature becoming low enough for inelastic interactions between the particles to stop. The net yield of all the particles gets fixed at this point. For some time, the particles still experience the elastic collision, and this elastic collision stops when the system expands enough, at the kinetic freeze-out. The particles’ interactions end at this stage and their transverse momentum spectra ($p_{T}$) get fixed. It should be noted that the freeze-out scenarios discussed above refer to kinetic decoupling, and we keep the focus of the present work on kinetic decoupling because we are studying the final state particles.

Indeed, the system evolution undergoes several stages, corresponding to different temperatures, as seen from the above discussion. The initial collision is the first stage of the system evolution, which corresponds to the initial temperature and describes the system’s characteristics at the initial stage. There is also another kind of temperature called the effective temperature, and it occurs just before the kinetic freeze-out temperature, which includes the flow effect. The details of these temperatures can be found in [17,18,19,20,21]. The present work is focused on the effective temperature, and we shall extract it from the transverse momentum spectra of the particles in different collisions.

The particles’ transverse momentum ($p_{T}$) spectra are essential because they give the particulars about [22] the transverse excitation degree and dynamic expansion of the collision system. This paper studied the identified and strange particles in Au–Au, Cu–Cu and Pb–Pb collisions at 62.4, 400 and 2760 GeV. We also analyzed the identical particles in pp collisions at the exact center of mass energy to compare the results of AA collisions with pp collisions.

The remainder of the paper consists of the method and formalism in Section 2, followed by the results and discussion in Section 3. In Section 4, we summarize our main observations and conclusions.

## 2. The Method and Formalism

It is believed that a few emission sources are formed in high-energy collisions according to the multithermal source model [23,24,25,26]. For nuclear fragments and for the other produced particles (such as identified, strange and charmed particles) from the target and projectile in nucleus–nucleus collisions, the sources for the former may be nucleon or nucleon clusters. In contrast, the seeds for the latter may be the participant quarks or gluons, although the contributors $c+\bar{c}$ can be from the gluon fusion. Different statistics such as Fermi–Dirac, Bose–Einstein, Boltzmann–Gibbs and Tsallis statistics can describe the properties of sources. The above statistics have relations with each other because they may result in similar or different distributions while describing the spectra of the produced hadrons.

The Boltzmann–Gibbs statistic describes the transverse momentum spectra of the particles in a narrow $p_{t}$ range, while the Tsallis statistic describes a wider $p_{T}$ range, although it is derived from the former [27,28,29]. In fact, the Boltzmann–Gibbs statistic is a special case of the Tsallis distribution in which entropy q=1. For the parameterization of the final state hadrons, the Tsallis distribution is widely used in high-energy collisions from lower to higher energies (such as from a few GeV to 13 TeV). The form of the Tsallis distribution [30,31,32,33,34,35,36] is expressed as

(1) $$\begin{array}{l} \frac{Ed^{3}N}{d^{3}p}=\frac{1}{2\pi p_{T}}\frac{d^{2}N}{dp_{T}dy}=\frac{dN}{dy}\frac{\left(n-1)(n-2\right)}{2\pi nT[nT+m_{0}\left(n-2\right)]}\\ \times {\left(1+\frac{m_{T}-m_{0}}{nT}\right)}^{-n} \end{array}$$

where *E* denotes the energy, and *p*, *N* and y are the momentum, number of particles and rapidity, respectively. $m_{T}$ is the transverse mass and can be represented as $m_{T}$ = $\sqrt{p_{T}^{2}+m_{0}^{2}}$ [37,38,39,40,41,42], and $m_{0}$ is the rest mass of the particle. *T* is the effective temperature, and *n* is the power index, particularly n=1/(q−1), where *q* describes the degree of equilibrium. The emission source is more equilibrated if q(n) is closer to 1 (it has a larger value).

Nonextensive thermodynamics is a new method for studying the heavy-ion collisions at relativistic energy. The Tsallis–Pareto function [42,43,44,45,46,47] can be used for fitting transverse momentum $\left(p_{T}\right)$ spectra in low as well as in intermediate regions, especially in the hadronization process, and demonstrates an impressive relation among hadrons. The $p_{T}$ distribution of the Tsallis–Pareto function can be expressed as

(2) $$\begin{array}{ll} f_{1}\left(p_{T}\right)= & \frac{1}{N}\frac{dN}{dp_{T}}=A\frac{\left(n-1)(n-2\right)}{nT[nT+m_{0}\left(n-2\right)]}\\ \; & \times {\left(1+\frac{m_{T}-m_{0}}{nT}\right)}^{-n} \end{array}$$

The present work is a continuation of our work published in [16,48,49,50,51,52,53,54,55,56] using different statistical fit functions to extract parameters relevant to the collective properties of the hadronic medium.

## 3. Results and Discussion

**Figure 1:**
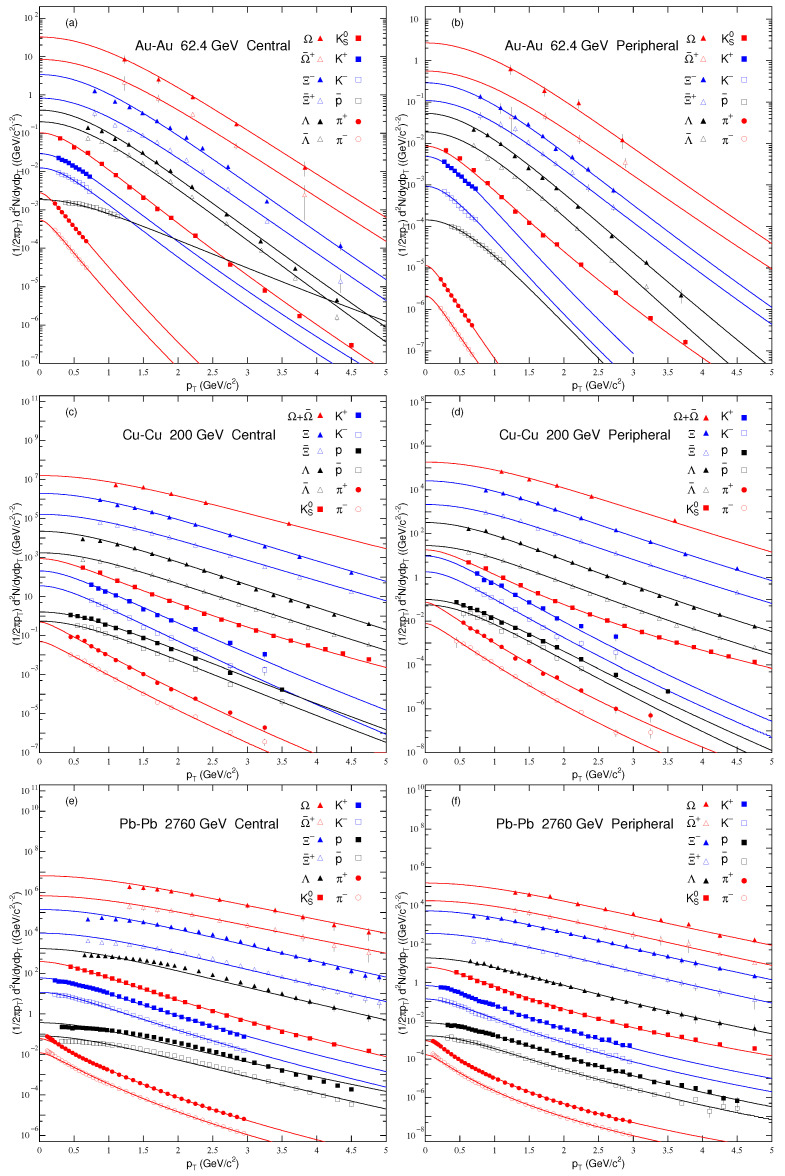
Transverse momentum spectra of nonstrange and strange particles produced in Au–Au, Cu–Cu and Pb–Pb collisions at 62.4 GeV in panels (**a**,**b**), at 200 GeV in panels (**c**,**d**) and at 2760 GeV in panels (**e**,**f**), respectively. The symbols represent the experimental data measured by the BRAHMS Collaboration [57], STAR Collaboration [29,58,59] and ALICE Collaboration [60,61]. The lines are the fits from Equation (2).

The transverse momentum spectra ($p_{T}$) of $\pi^{+}$, $\pi^{-}$, $K^{+}$, $K^{-}$, *p*, $\bar{p}$, $K_{s}^{0}$, Λ, Ξ or ${\bar{\Xi }}^{+}$ and Ω or ${\bar{\Omega }}^{+}$ or $\Omega +\bar{\Omega }$ produced in the most central and peripheral nucleus–nucleus collisions are displayed in Figure 1. Panels (a) and (b) show the $p_{T}$ spectra of the nonstrange and strange particles in Au−Au collisions at $\sqrt{s_{NN}}=62.4$ GeV, while panels (c) and (d) show the $m_{T}$ spectra of these mentioned particles in Cu–Cu collisions at $\sqrt{s_{NN}}=200$ GeV. Panels (e) and (f) represent the $p_{T}$ spectra of nonstrange and strange particles in Pb–Pb collisions at $\sqrt{s_{NN}}=2760$ GeV. The rapidity for $\pi^{+}$, $\pi^{-}$, $K^{+}$, $K^{-}$, *p* and $\bar{p}$ in panels (a) and (b) is |y|<0.1. Similarly, for $K_{s}^{0}$, $\bar{\Lambda }$, Λ, ${\bar{\Xi }}^{+}$, $\Xi^{-}$, ${\bar{\Omega }}^{+}$ and Ω, |y|<0.1. Similarly, the rapidity for all the particles, as mentioned earlier in panels (c)–(f) is |y|<0.5. The symbols are used to display the experimental data from the BRAHMS Collaboration [57], STAR Collaboration [29,58,59] and ALICE Collaboration [60,61], and the curves over the data are our fit results by using the Tsallis–Pareto type function. It can be seen that Equation (2) fits the data approximately well. Different symbols represent different particles. The filled and open symbols show the positive and negative charged particles.

**Figure 2:**
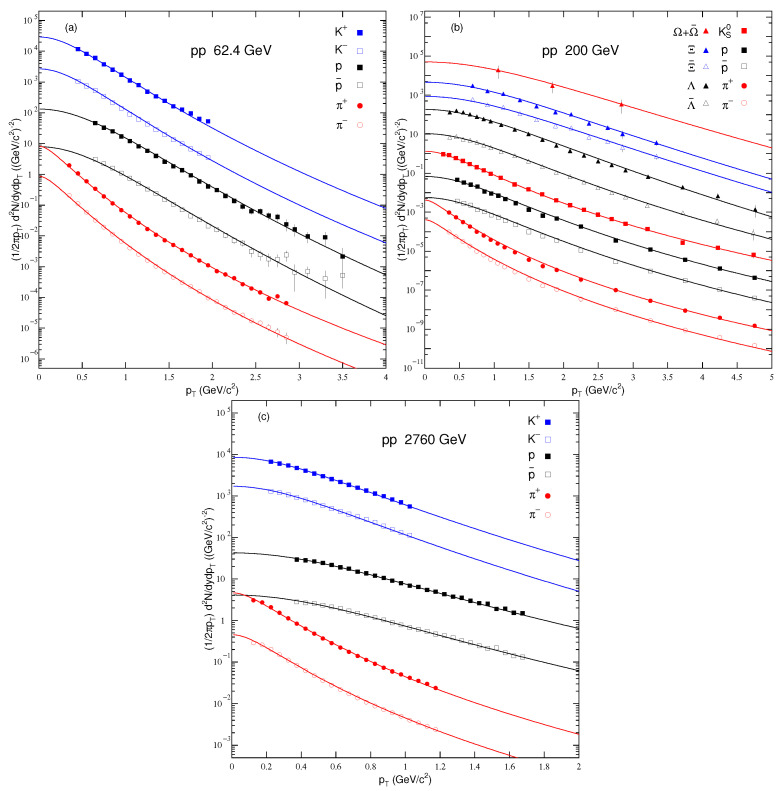
Transverse momentum spectra of identified and strange particles in pp collisions at (**a**) 62.4, (**b**) 200 and (**c**) 2760 GeV.The symbols represent the experimental data measured by the PHENIX Collaboration [37], STAR Collaboration [29,38,62] and CMS Collaboration [42]. The lines are the fits from Equation (2).

Figure 2 is similar to Figure 1, but it shows the transverse momentum spectra of the particles in p–p collisions at 62.4, 200 and 2760 GeV in panels (a)–(c), respectively. Panel (a) presents the transverse momentum spectra of $\pi^{+}$, $\pi^{-}$, $K^{+}$, $K^{-}$, *p* and $\bar{p}$, while panel (b) presents the transverse momentum spectra of $\pi^{+}$, $\pi^{-}$, $K^{+}$, $K^{-}$, *p*, $\bar{p}$, $K_{s}^{0}$, $\bar{\Lambda }$, Λ, $\bar{\Xi }$, Ξ and $\Omega +\bar{\Omega }$, and panel (c) displays the transverse momentum spectra of $\pi^{+}$, $\pi^{-}$, $K^{+}$, $K^{-}$, *p* and $\bar{p}$. Different symbols represent different particles. The symbols are used to display the experimental data from the PHENIX Collaboration [37], STAR Collaboration [29,38,62] and CMS Collaboration [42], and the curves over the data are our fit results by Equation (2). The filled and open symbols show the positive and negative charged particles, respectively. The related extracted parameters, along with $\chi^{2}$ and degree of freedom (DOF) are listed in Table 1. One can see that Equation (2) fits the data well in pp collisions at 62.4, 200 and 2760 GeV at the RHIC and LHC.

**Figure 3:**
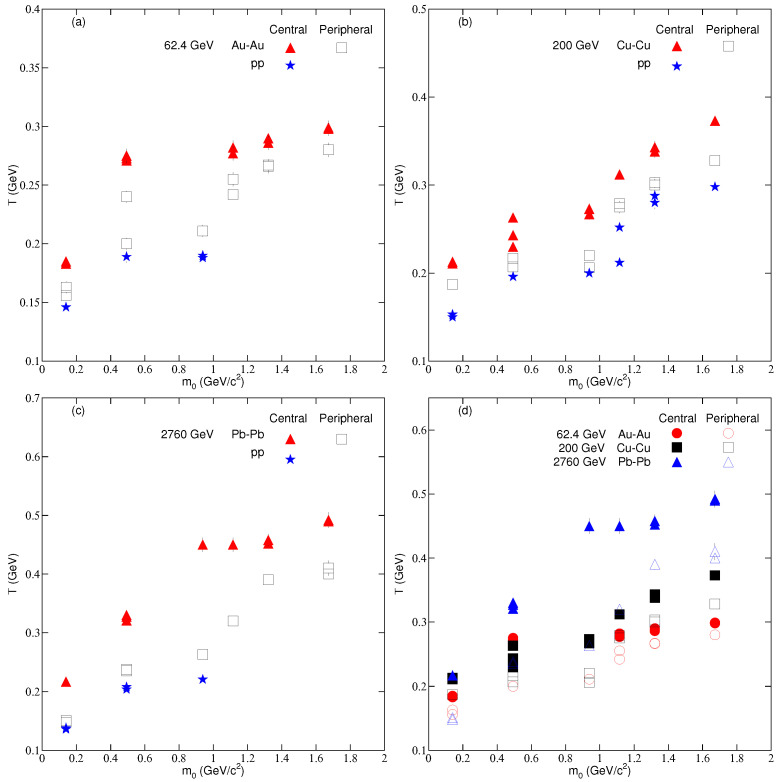
Dependence of the effective temperature on centrality and mass of the particles in nucleus–nucleus and pp collisions at (**a**) 62.4 GeV (**b**) 200 GeV (**c**) 2760 GeV and (**d**) (62.4, 200, and 2760) GeV energies.

Figure 3 shows the result of the dependence of *T* on $m_{0}$ and centrality. Different symbols are used to represent different collisions. Filled and empty symbols show the central and peripheral collisions, respectively, and the blue-colored star symbols denote the pp collisions. The symbols from left to right show the mass dependence of the parameters. One can see that *T* is slightly larger in the central collisions compared to the peripheral collisions because there is a large number of participants in the former, which makes the reaction very intense, and thus more energy is stored in the former. These results validate our recent results [16,18,48,49]. In addition, *T* in pp collisions are also shown, which is slightly lower than or approximately equal to that in peripheral AA collisions at the same center of mass energy. We also note that *T* in AA and pp collisions increases with increasing $m_{0}$, which shows a differential freeze-out scenario that validates our previous results [17,20,53,54,56]. *T* is the temperature which includes the contribution of both the kinetic freeze-out temperature and radial flow; therefore, the freeze-out refers to the kinetic freeze-out. Different *T*s for different particles indicate that the scenario of the decoupling of the particles is a multiple-kinetic-decoupling scenario. In the present work, different collision systems with different center of mass energies were considered to check the system size and energy dependence of *T*. Still, we did not report any specific dependence of *T* on either.

**Figure 4:**
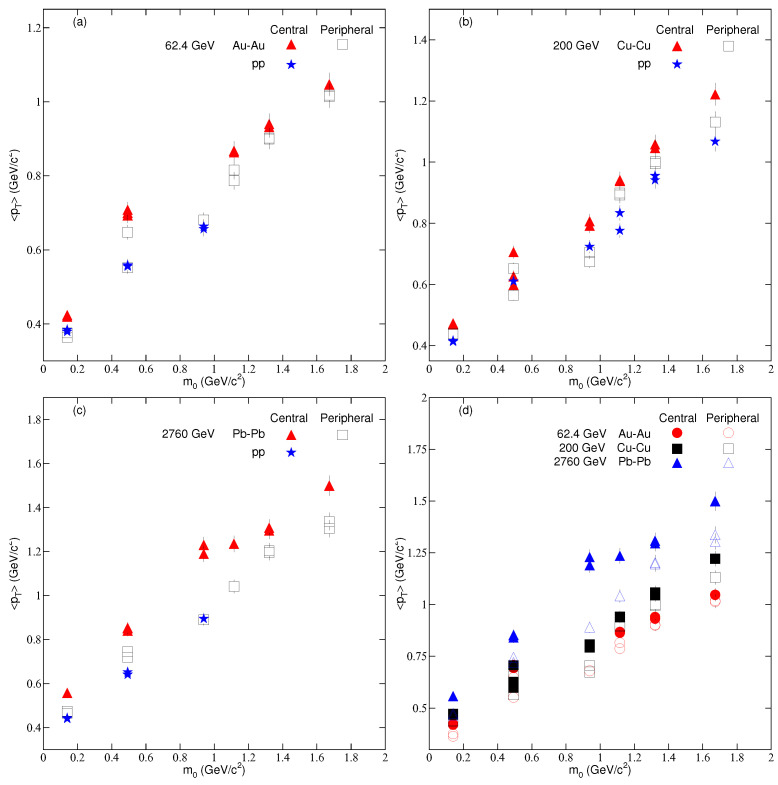
Dependence of the mean transverse momentum on centrality and mass of the particles in nucleus–nucleus and pp collisions at (**a**) 62.4 GeV (**b**) 200 GeV (**c**) 2760 GeV and (**d**) (62.4, 200, and 2760) GeV.

Figure 4 is similar to Figure 3, but it displays the dependence of the mean transverse momentum ($<p_{T}>$) on $m_{0}$ and centrality. We note that $<p_{T}>$ is slightly larger in a central collision than in peripheral and pp collisions. This is because more energy is transported in the system in central collisions than in the latter two. $<p_{T}>$ in pp collisions is close to that in peripheral AA collisions at the same center of mass energy. $<p_{T}>$ also depends on $m_{0}$. The heavier the particle, the larger the $<p_{T}>$. We can see that $<p_{T}>$ is larger in Pb–Pb collisions than in Au–Au and Cu–Cu collisions, which shows that $<p_{T}>$ depends on the size of the system, but this dependence is weak because the values of *T* in the Au–Au and Cu–Cu collisions are approximately close to each other due to the different collision energies of the two systems. The Au−Au system is approximately three times larger than the Cu–Cu system, but its collision energy is approximately three times lower than that of Cu–Cu collisions, and this may increase the energy dependence of $<p_{T}>$, which becomes more prominent in pp collisions because we can see that $<p_{T}>$ is larger at 2760 GeV than at 200 GeV, and $<p_{T}>$ at the latter is larger than at 62.4 GeV.

**Figure 5:**
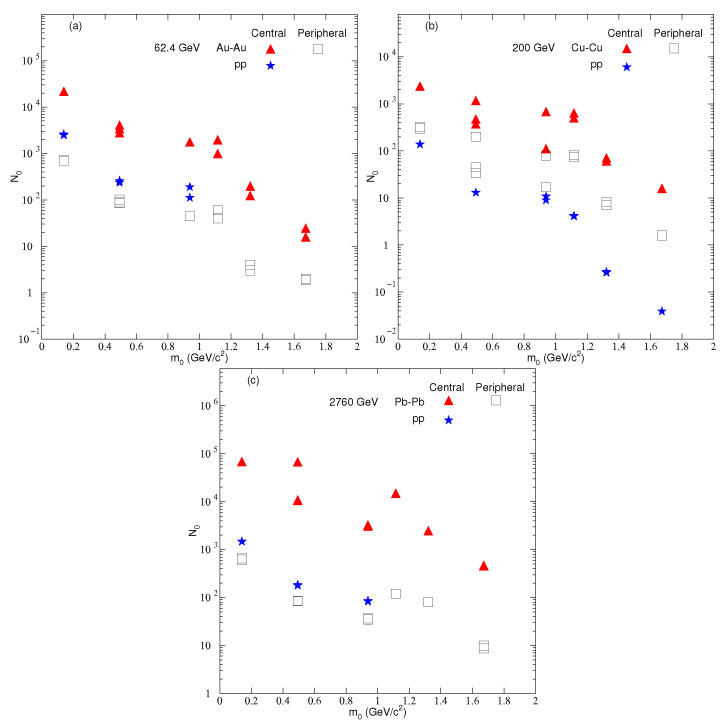
Dependence of the mean $N_{0}$ on centrality and mass of the particles in nucleus–nucleus and pp collisions at (**a**) 62.4 GeV (**b**) 200 GeV (**c**) 2760 GeV.

Figure 5 is similar to Figure 3, but it shows the dependence of $N_{0}$ on $m_{0}$ and centrality. $N_{0}$ is the multiplicity parameter, not only the normalization constant. It can be seen that $N_{0}$ is slightly larger in central AA collisions than in peripheral AA collisions as well as pp collisions, since the central collision systems are larger and more violent than the latter two collisions, which results in an enormous multiplicity. In most cases, it is also observed that $N_{0}$ in pp collisions is close to the peripheral AA collisions at the exact center of mass energy. $N_{0}$ is reported to be mass-dependent. The heavier the particles, the smaller the multiplicity. However, $N_{0}$’s dependence on the size of the system in central AA collisions can be seen. The larger the size of the system is, the larger the $N_{0}$.

**Figure 6:**
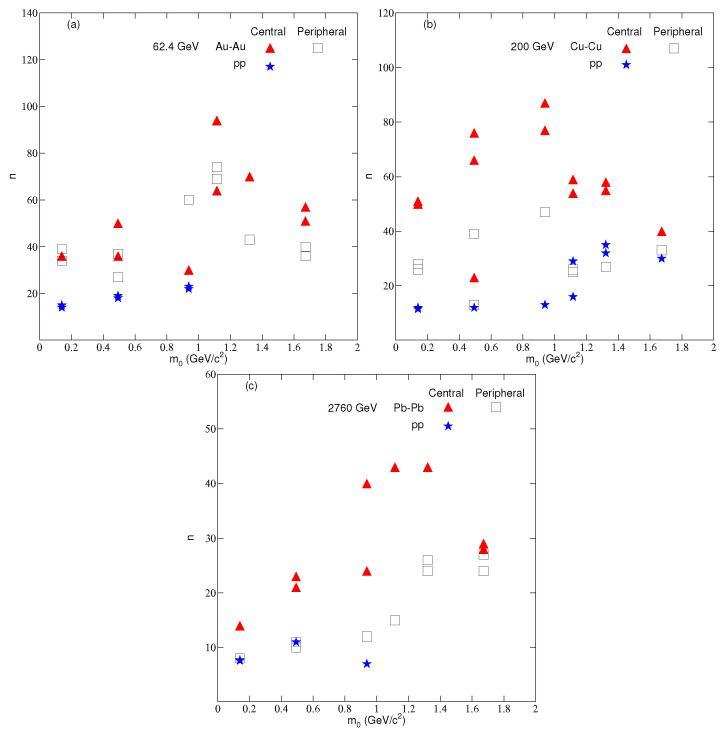
Dependence of the mean *n* on centrality and mass of the particles in nucleus–nucleus and pp collisions at (**a**) 62.4 GeV (**b**) 200 GeV (**c**) 2760 GeV.

Figure 6 is similar to Figure 5, but it represents the result for the entropy parameter *n*. As discussed in the second section, *n* measures the degree of equilibrium of the system. The larger the value of *n*, the closer the system will be to an equilibrium state. Figure 6 highlights that *n* is higher in most cases in central collisions than in peripheral collisions and *pp* collisions, which means that the central collision system equilibrates quickly.

Before going to the conclusion section, we would like to point out that the central collision has a more significant *T* and *n*. The central collision system approaches the equilibrium state quickly compared to the peripheral and pp collisions. However, in peripheral collisions, the system has a lower *T* and *n*, away from the equilibrium state.

## 4. Conclusions

The main observations and conclusions are summarized here.

(a) The transverse momentum spectra of identified and strange particles were analyzed in Au-Au, Cu-Cu and Pb–Pb collisions at 62.4 GeV, 200 GeV and 2760 GeV, respectively, by the Tsallis–Pareto type function, and the effective temperature and mean transverse momentum were extracted. We also analyzed the pp collisions at 62.4 GeV, 200 GeV and 2760 GeV to check the nature of the extracted parameters in the peripheral AA collisions and pp collisions at the exact center of mass energy.
(b) The effective temperature (*T*) was more prominent in a central collision than in a peripheral collision because many hadrons were involved in the reaction, which transferred more energy in the central collision systems. *T* in peripheral collisions was closer to that of pp collisions at the exact center of mass energy, which showed that the two systems had similar thermodynamic properties.
(c) The mean transverse momentum was more significant in central collisions than in peripheral collisions due to substantial momentum transfer. In peripheral collisions, it was close to that of the pp collisions.
(d) Both the effective temperature and mean transverse momentum were mass-dependent and increased with mass. The increase of *T* with $m_{0}$ was consistent with the multiple kinetic freeze-out scenarios.
(e) $<p_{T}>$ was larger in Pb–Pb collisions than in Au–Au and Cu–Cu collisions, and in the latter two cases, the values were close to each other, which showed a weak dependence on the size of the system and comparatively strong dependence on the collision’s energy because it increased with the increase of energy in pp collisions.
(f) The multiplicity parameter $N_{0}$ was slightly larger in central AA collisions than in peripheral AA collisions. In peripheral collisions, it was close to that in pp collisions at the exact center of mass energy. In addition, $N_{0}$ was mass-dependent and was higher for lighter particles. $N_{0}$ in central AA collisions depended on the size of the interacting system; larger sizes of the interacting system yielded higher values of the $N_{0}$.
(g) The entropy parameter *n* was larger in a central collision, rendering the system to an equilibrium state more quickly compared to the peripheral collisions.

## Figures and Tables

**Table 1:** Values of free parameters *T* and *n*, normalization constant ($N_{0}$), mean transverse momentum ($<p_{T}>$), $\chi^{2}$ and degree of freedom (dof) corresponding to the curves in Figure 1 and Figure 2.

Figure	Collab.	Centrality	Particle	Factor	*T* (GeV)	*n*	$<p_{T}>$	$N_{0}$	$\chi^{2}$	dof
Figure 1a	STAR	0–5%	$\pi^{+}$	0.000005	0.183±0.004	36±2	0.420±0.013	21,980 ± 14	28	7
Au-Au			$\pi^{-}$	0.000001	0.185±0.003	36±1	0.424±0.013	21,980 ± 22	25	7
62.4			$K^{+}$	0.001	0.275±0.006	36±2	0.708±0.021	4100±13	1	7
			$K^{-}$	0.0005	0.271±0.003	36±1	0.700±0.021	3410±32	12	7
			$\bar{p}$	0.0005	0.501±0.008	30±0.5	1.316±0.039	1790±14	165	13
			Λ	0.05	0.277±0.006	64±2	0.863±0.026	1996±14	103	9
			$\bar{\Lambda }$	0.05	0.282±0.006	94±4	0.867±0.026	1009±13	84	9
			$\Xi^{-}$	5	0.286±0.006	70±4	0.931±0.028	200±9	62	8
			${\bar{\Xi }}^{+}$	2	0.290±0.003	70±0.1	0.940±0.028	124±3	39	8
			$K_{S}^{0}$	0.005	0.273±0.006	50±2	0.693±0.021	2800±12	154	12
		0–20%	Ω	500	0.298±0.006	51±2	1.047±0.031	25±0.3	1	2
			${\bar{\Omega }}^{+}$	200	0.299±0.006	57±2	1.046±0.031	16±1	3	2
Figure 1b	STAR	70–80%	$\pi^{+}$	0.0000005	0.156±0.003	34±2	0.364±0.011	707±13	42	7
			$\pi^{-}$	0.0000001	0.163±0.005	39±2	0.375±0.011	697±5	32	7
			$K^{+}$	0.005	0.200±0.004	37±2	0.552±0.017	89±6	10	7
			$K^{-}$	0.001	0.200±0.005	37±2	0.552±0.017	85±2	16	7
			$\bar{p}$	0.0005	0.211±0.005	60±3	0.681±0.020	45±2	14	13
		60–80%	Λ	0.2	0.255±0.006	69±4	0.815±0.024	60±5	27	8
			$\bar{\Lambda }$	0.1	0.242±0.004	74±3	0.787±0.024	40±2	20	7
			$\Xi^{-}$	20	0.266±0.006	43±2	0.900±0.027	4±0.07	4	6
			${\bar{\Xi }}^{+}$	10	0.267±0.005	43±1	0.902±0.027	3±0.06	7	6
			$K_{S}^{0}$	0.01	0.240±0.005	27±0.8	0.648±0.019	102±2	46	11
		40–60%	Ω	500	0.280±0.006	40±3	1.014±0.030	2±0.007	2	1
			${\bar{\Omega }}^{+}$	100	0.280±0.004	36±1	1.018±0.031	2±0.03	10	1
Figure 1c	BRAHMS	0–10%	$\pi^{+}$	0.01	0.211±0.005	50±2	0.469±0.014	2370±21.00	34	11
Cu-Cu			$\pi^{-}$	0.001	0.213±0.005	51±3	0.473±0.014	2380±18.00	34	11
200			$K^{+}$	50	0.243±0.005	66±2	0.627±0.019	475±10.00	19	8
			$K^{-}$	10	0.230±0.005	76±2	0.598±0.018	376±7.00	27	8
			*p*	0.5	0.273±0.006	77±4	0.806±0.024	683±9.00	47	11
			$\bar{p}$	1	0.267±0.006	87±2	0.792±0.024	112±4.00	48	10
	STAR		$K_{S}^{0}$	100	0.263±0.005	23±0	0.706±0.021	1180±12.00	186	15
			Λ	10,000	0.312±0.005	59±2	0.939±0.028	640±9.00	118	16
			Ξ	10,000,000	0.338±0.007	58±2	1.046±0.031	71±3.00	11	8
			$\bar{\Xi }$	1,000,000	0.343±0.007	55±2	1.058±0.032	61±3.00	17	8
			$\bar{\Lambda }$	1000	0.312±0.005	54±2	0.941±0.028	505±11.00	54	16
			$\Omega +\bar{\Omega }$	500,000,000	0.373±0.005	40±2	1.222±0.037	16±0.60	27	2
Figure 1d	BRAHMS	50–70%	$\pi^{+}$	0.01	0.187±0.003	26±0	0.440±0.013	299±7.00	24	11
			$\pi^{-}$	0.001	0.187±0.004	28±0	0.437±0.013	320±8.00	11	11
			$K^{+}$	20	0.207±0.005	39±1	0.565±0.017	45±2.00	14	7
			$K^{-}$	5	0.207±0.004	39±2	0.565±0.017	34±1.00	7	8
			*p*	0.2	0.220±0.005	47±2	0.705±0.021	80±6.00	28	11
			$\bar{p}$	0.5	0.206±0.005	47±2	0.674±0.020	17±0.05	19	8
	STAR	40–60%	$K_{S}^{0}$	10	0.217±0.004	13±0	0.652±0.020	199±6.00	40	15
			Λ	1000	0.275±0.007	25±1	0.892±0.027	82±3.00	52	16
			$\bar{\Lambda }$	100	0.279±0.005	26±1	0.899±0.027	73±3.00	33	16
			Ξ	1,000,000	0.300±0.007	27±0	0.995±0.030	8±0.01	10	8
			$\bar{\Xi }$	100,000	0.303±0.006	27±1	1.002±0.030	7±0.00	10	8
			$\Omega +\bar{\Omega }$	50,000,000	0.328±0.005	33±1	1.131±0.034	2±0.01	15	2
Figure 1e	ALICE	0–5%	$\pi^{+}$	0.00005	0.217±0.006	14±0	0.558±0.017	68,960 ±32	63	38
Pb-Pb			$\pi^{-}$	0.00001	0.217±0.006	14±1	0.558±0.017	68,360 ± 33	53	38
2760			$K^{+}$	1	0.327±0.006	21±0	0.853±0.026	10,900 ± 22	35	33
			$K^{-}$	0.2	0.330±0.007	23±0	0.851±0.026	10,680 ± 30	23	33
			*p*	0.05	0.450±0.011	24±0	1.229±0.037	3083±32	419	39
			$\bar{p}$	0.01	0.450±0.012	40±1	1.189±0.036	3240±28	128	39
			$K_{S}^{0}$	1	0.321±0.007	21±0	0.840±0.025	67,800 ± 23	20	25
			Λ	50	0.450±0.012	43±2	1.235±0.037	15,100 ± 26	122	23
		0–10%	$\Xi^{-}$	30,000	0.458±0.010	43±2	1.308±0.039	2500±13	136	19
			${\bar{\Xi }}^{+}$	2000	0.452±0.007	43±3	1.295±0.039	2508±16	104	19
			Ω	1,000,000	0.490±0.009	28±0	1.499±0.045	470±13	4	8
			${\bar{\Omega }}^{+}$	10,000,000	0.492±0.013	29±0	1.500±0.045	460±10	4	8
Figure 1f	ALICE	80–90%	$\pi^{+}$	0.00005	0.148±0.005	8±0	0.466±0.014	650±12	82	38
			$\pi^{-}$	0.00001	0.151±0.004	8±0	0.475±0.014	621±21	68	38
			$K^{+}$	1	0.235±0.004	11±0	0.719±0.022	86±3	23	33
			$K^{-}$	0.2	0.235±0.006	11±0	0.719±0.022	86±5	35	33
			*p*	0.05	0.263±0.007	12±0	0.890±0.027	35±0.9	14	39
			$\bar{p}$	0.01	0.263±0.006	12±0	0.890±0.027	37±0.6	26	39
			$K_{S}^{0}$	10	0.238±0.007	10±0	0.744±0.022	83±3	1	25
			Λ	50	0.320±0.005	15±0	1.041±0.031	120±6	12	26
		60–80%	$\Xi^{-}$	30,000	0.390±0.008	24±0	1.203±0.036	80±3	11	16
			${\bar{\Xi }}^{+}$	2000	0.390±0.007	26±1	1.196±0.036	80±3	35	16
			Ω	1,000,000	0.400±0.008	27±0	1.304±0.039	10±0.06	1	7
			${\bar{\Omega }}^{+}$	10,000,000	0.410±0.013	24±0	1.337±0.040	9±0.009	2	7
Figure 2a	PHENIX		$\pi^{+}$	0.1	0.146±0.002	14±0.040	0.384±0.012	2500±31	32	23
pp			$\pi^{-}$	0.01	0.146±0.003	15±0.100	0.379±0.011	2600±0	26	23
62.4			$K^{+}$	1	0.189±0.005	18±0.050	0.559±0.017	259±7	14	13
			$K^{-}$	1000	0.189±0.003	19±0.030	0.555±0.017	237±9	20	13
			*p*	100	0.190±0.002	22±0.080	0.663±0.020	190±7	17	24
			$\bar{p}$	10	0.188±0.003	23±0.050	0.656±0.020	112±6	24	24
Figure 2b	STAR		$\pi^{+}$	0.001	0.150±0.004	12±0.001	0.413±0.012	138±6	72	16
pp			$\pi^{-}$	0.0001	0.153±0.004	12±0.050	0.416±0.012	138±2	86	16
200			*p*	1	0.200±0.002	13±0.070	0.723±0.022	10.80±0.003	31	15
			$\bar{p}$	0.1	0.200±0.005	13±0.080	0.723±0.022	9.00±0.004	56	15
			$K_{S}^{0}$	10	0.196±0.002	12±0.009	0.610±0.018	13.00±0.040	14	19
			Λ	10,000	0.252±0.006	29±0.050	0.834±0.025	4.20±0.0030	82	18
			$\bar{\Lambda }$	500	0.212±0.004	16±0.070	0.776±0.023	4.10±0.0030	29	18
			Ξ	5,000,000	0.280±0.007	32±0.050	0.942±0.028	0.27±0.0005	8	8
			$\bar{\Xi }$	1,000,000	0.288±0.004	35±0.200	0.956±0.029	0.26±0.0007	9	8
			$\Omega +\bar{\Omega }$	500,000,000	0.298±0.005	30±0.700	1.068±0.032	0.039±0.0004	0	0
Figure 2c	CMS		$\pi^{+}$	0.1	0.136±0.003	8±0.001	0.440±0.013	1480±14	40	19
pp			$\pi^{-}$	0.01	0.138±0.002	8±0.008	0.444±0.013	1470±15	43	19
2760			$K^{+}$	5000	0.208±0.006	11±0.008	0.651±0.020	184±6	26	14
			$K^{-}$	1000	0.204±0.005	11±0.008	0.641±0.019	179±5	63	14
			*p*	100	0.221±0.007	7±0.080	0.894±0.027	86±3	56	24
			$\bar{p}$	10	0.221±0.004	7±0.040	0.894±0.027	83±2	77	24

## Data Availability

The data used to support the findings of this study are included within the article and are cited at relevant places within the text as references.
